# Reconstruction After an Anterior Cruciate Ligament Injury Does Not Reduce the Osteoarthritis Risk But Lowers the Total Knee Arthroplasty Rate: A Systematic Review and Meta-analysis

**DOI:** 10.1177/23259671261451241

**Published:** 2026-07-30

**Authors:** Benjamin Blackman, Matey Juric, Seth Ybema, Marc Daniel Bouchard, Matthew Macciacchera, Bogdan A. Matache, Jihad Abouali

**Affiliations:** * Division of Orthopaedic Surgery, University of Toronto, Toronto, Ontario, Canada; † Faculty of Medicine, University of Ottawa, Ottawa, Ontario, Canada; ‡ Michael G. DeGroote School of Medicine, McMaster University, Hamilton, Ontario, Canada; § Division of Orthopaedic Surgery, Department of Surgery, McMaster University, Hamilton, Ontario, Canada; ‖ Division of Orthopaedic Surgery, University of Ottawa, Ottawa, Ontario, Canada; ¶ Department of Surgery, The Ottawa Hospital, Ottawa, Ontario, Canada; Investigation performed at University of Toronto, Toronto, Ontario, Canada

**Keywords:** anterior cruciate ligament, reconstruction, nonoperative, osteoarthritis, long-term outcomes

## Abstract

**Background:**

An anterior cruciate ligament (ACL) rupture is a common and debilitating knee injury. Although surgical management restores knee stability, its effectiveness in preventing long-term joint degeneration compared with nonoperative management remains unclear.

**Purpose/Hypothesis:**

The purpose of this study was to compare the long-term incidence of radiographic knee osteoarthritis (OA) after the operative versus nonoperative management of ACL injuries. It was hypothesized that operative management would lead to lower rates of OA.

**Study Design:**

Systematic review and meta-analysis; Level of evidence, 3.

**Methods:**

A systematic review and meta-analysis were conducted per PRISMA (Preferred Reporting Items for Systematic Reviews and Meta-Analyses) guidelines. PubMed, Embase, and Scopus were searched through June 2025 for comparative studies evaluating the development of OA after an ACL injury that was treated with operative or nonoperative management. Radiographic OA was defined as Kellgren-Lawrence grade ≥2 or equivalent. Random-effects meta-analyses were performed to compare radiographic OA, secondary meniscal surgery, and total knee arthroplasty (TKA) rates between treatment groups. Study quality was assessed using the MINORS (Methodological Index for Non-Randomized Studies) instrument and the Cochrane RoB 2 (risk of bias tool). The certainty of evidence was assessed using the GRADE (Grading of Recommendations Assessment, Development and Evaluation) framework.

**Results:**

A total of 18 studies comprising 33,540 patients (mean follow-up, 12.3 years) were included. A meta-analysis of 15 studies found no significant difference in radiographic OA between the ACL reconstruction (ACLR) and nonoperative groups (risk ratio [RR], 1.01 [95% CI, 0.79-1.27]; *I*^2^ = 92%; *P* = .96). A sensitivity analysis of level 1 to 2 studies with low heterogeneity yielded similar findings (RR, 1.14 [95% CI, 0.87-1.48]; *I*^2^ = 34%; *P* = .33). A meta-analysis of 2 studies assessing OA prevalence in the ACL repair versus nonoperative group also found no statistically significant difference, with moderate heterogeneity (RR, 0.78 [95% CI, 0.54-1.12]; *I*^2^ = 55%; *P* = .18). Rates of secondary meniscal surgery did not differ significantly (RR, 0.79 [95% CI, 0.52-1.21]; *I*^2^ = 56%; *P* = .29). However, ACLR significantly reduced the risk of TKA (RR, 0.17 [95% CI, 0.07-0.43]; *I*^2^ = 59%; *P* = < .001).

**Conclusion:**

The operative management of ACL injuries did not decrease the long-term risk of developing radiographic OA compared with nonoperative management. ACLR was associated with a lower rate of TKA.

An anterior cruciate ligament (ACL) rupture is a common and debilitating knee injury that disrupts joint stability and places patients at an increased risk for long-term degenerative changes.^15^ It typically occurs during sports or high-demand physical activity involving sudden deceleration, pivoting, or poor landing mechanics and is often accompanied by a concomitant meniscal or chondral injury.^22,60^ Biomechanically, the loss of ACL integrity alters tibiofemoral kinematics and increases shear forces across the articular cartilage, predisposing patients to early-onset posttraumatic osteoarthritis (OA).^10,54,63^ Rates of OA after an ACL injury vary widely in the literature, with studies reporting 0% to 100% within 10 years.^6,35,54^

Management strategies for an ACL rupture typically involve operative (reconstruction or repair) or nonoperative (physical therapy) interventions.^44,47^ Currently, ACL reconstruction (ACLR) is the gold standard in operative management.^12,24,56^ ACLR aims to restore mechanical stability, reduce recurrent instability, and prevent secondary injuries to meniscal and chondral structures.^12^ Nonoperative treatment, which typically consists of structured physical therapy, focuses on neuromuscular retraining, strengthening, and activity modification to achieve functional stability without surgery.^49,53^ The decision between operative versus nonoperative treatment remains debated, with each carrying theoretical benefits and risks for long-term joint health.

Operative management aims to protect against further meniscal damage and recurrent instability while allowing for the treatment of associated injuries such as meniscal, chondral, and collateral ligament lesions, which may influence long-term joint outcomes. However, it may introduce the risk of iatrogenic trauma and the potential for graft failure, which may contribute to OA progression.^59^ Conversely, nonoperative management avoids these risks while possibly allowing patients to achieve a knee joint that they are functionally satisfied with.^40,43,45^

Despite the clinical importance of this question, clear evidence regarding whether operative management reduces the risk of knee OA compared with nonoperative treatment is lacking. Prior studies have reported conflicting findings, with some demonstrating higher rates of OA after reconstruction, others showing no difference, and some suggesting a protective effect of surgery.^7,37,68^ These inconsistencies are likely driven by the heterogeneity in OA definitions, imaging modalities, patient selection, study period, surgical techniques for ACLR, and duration of follow-up. As a result, the true effect of operative versus nonoperative management on long-term radiographic OA remains uncertain.^16,50^

This systematic review and meta-analysis synthesized available evidence, comparing the incidence of radiographic knee OA after the operative versus nonoperative management of ACL injuries. We hypothesized that operative management would lead to lower rates of OA compared to nonoperative management. We aimed to examine this hypothesis by emphasizing a long-term follow-up and standardized radiographic criteria.

## Methods

This systematic review was conducted according to the Preferred Reporting Items for Systematic Reviews and Meta-Analyses (PRISMA) guidelines.^34^ Institutional review board approval was not required, as this study consists of only previously published data.

### Search Strategy

There were 3 online databases (Embase, Scopus, and PubMed) searched from inception to June 2, 2025, for studies discussing ACL injuries treated with surgery or nonoperative management as well as the subsequent development or progression of OA. AppendixTable A1 outlines the complete search terms and strategy. Studies were included if they (1) assessed ACL injuries in human participants of any age; (2) compared surgical interventions (reconstruction or repair) with nonoperative management; and (3) reported clinical, radiographic, or diagnostic outcomes of OA at any follow-up length. Studies were excluded if they (1) were noncomparative studies or secondary literature, such as case reports, reviews, or editorials, and (2) did not report objective outcomes related to OA. Only studies published in English or with available English translations were included.

### Study Screening

Screening of titles and abstracts was conducted independently by 3 authors (B.B., M.J., S.Y.). Disagreements regarding study inclusion were resolved by a consensus among the same assessors. When a consensus could not be reached, a fourth author (M.D.B.) was consulted. A full-text review was then conducted likewise.

### Quality Assessment

The methodological quality of nonrandomized studies was evaluated using the Methodological Index for Non-Randomized Studies (MINORS) criteria.^57^ Using the items on the MINORS checklist, noncomparative studies can achieve a maximum score of 16, while comparative studies can achieve a maximum score of 24. Noncomparative studies were categorized a priori as follows: 0-4 indicated very low quality, 5-7 indicated low quality, 8-12 indicated fair quality, and ≥13 indicated high quality. For comparative studies, categorization was determined a priori as follows: 0-6 indicated very low quality, 7-10 indicated low quality, 11-15 indicated fair quality, 16-20 indicated good quality, and >20 indicated high quality. The quality assessment of randomized controlled trials (RCTs) was conducted using the Cochrane RoB 2 (risk of bias tool).^61^ There were 5 domains evaluated: (1) bias from the randomization process, (2) bias due to deviations from intended interventions, (3) bias due to missing outcome data, (4) bias in measurement of the outcome, and (5) bias in selection of the reported result. Each domain was categorized as either (1) low risk of bias, (2) some concerns, or (3) high risk of bias. The overall risk of bias was considered to be “low risk” for studies assessed as having a low risk of bias in all domains, “some concerns” if the study raised some concerns in at least one domain, and “high risk” if the study was judged as having a high risk of bias in at least one domain or if the study raised some concerns in multiple domains. The quality assessment was performed in duplicate, and the mean calculated score was reported.

The certainty of evidence for each outcome was assessed using the Grading of Recommendations Assessment, Development and Evaluation (GRADE) framework by 2 independent reviewers (M.J. and S.Y.). Evidence was evaluated across 5 domains: risk of bias, inconsistency, indirectness, imprecision, and publication bias. RCTs were initially rated as having high-certainty evidence, while observational studies were assigned a starting rating of low certainty. The certainty of evidence was downgraded based on concerns within the 5 domains and upgraded when appropriate, such as in the presence of a large magnitude of effect. Overall certainty was categorized as high, moderate, low, or very low. A Summary of Findings table was generated using the GRADEpro Guideline Development Tool to present the certainty of evidence and effect estimates for each outcome (AppendixTable A2).

### Data Extraction

Data extraction from included articles was performed by 3 authors (B.B., M.J. and S.Y.), with data compiled using Google Sheets. Patient information was collected, including sample size, sex, mean age, follow-up time, associated injuries and procedures, and loss to follow-up. Early ACLR referred to surgery performed within the acute postinjury period, as defined by each study, while delayed ACLR referred to surgery performed after an initial period of nonoperative management. Outcomes included symptomatic OA; radiographic OA; OA grade; the need for meniscal repair, ACLR, or total knee arthroplasty (TKA) after initial management; and adverse events.

### Definition of Radiographic OA

Radiographic OA was defined a priori to ensure consistency across different classification systems used in the included studies. When the Kellgren-Lawrence (KL) scale was used, OA was considered present for KL grade ≥2, corresponding to definite osteophytes with possible or definite joint space narrowing. KL grade 1 was excluded from the primary definition. For studies using the Fairbank classification, only moderate or severe changes were categorized as OA, as “slight” changes align more closely with KL grade 1 and are not typically regarded as established disease. When the International Knee Documentation Committee (IKDC) radiographic grading system was used, OA was defined as grades B to D, encompassing small osteophytes and <25% narrowing (grade B), 25% to 50% narrowing (grade C), and >50% narrowing with sclerosis or a deformity (grade D). Grade A (normal) was considered no OA. In studies reporting OA severity descriptively as mild, moderate, or severe, all 3 categories were incorporated into the prevalence estimate, consistent with the KL grade ≥2 definition. AppendixTable A3 summarizes the OA definitions used in each included study and their corresponding classification within the standardized framework applied in this review.

### Statistical Analysis

Meta-analyses were conducted using a random-effects model (Python [Version 3.8.10] and DataParty; Python Software Foundation). The risk ratio (RR) and 95% confidence interval (CI) were calculated. When studies did not report the standard deviation, it was estimated using the following formula: range/4.^25^ The *I*^2^ statistic was used to assess heterogeneity. *I*^2^ values of 25%-49% were considered low, 50%-74% were considered moderate, and ≥75% were considered to be high statistical heterogeneity.^23^ When heterogeneity was considered high, pooled estimates were not interpreted.^20,39^ Other results from the studies were reported descriptively. Google Sheets was used to summarize means, ranges, and percentages. Studies that performed subgroup analyses and found statistical significance were reported with *P* values included. Statistical significance was defined as *P* < .05.

## Results

### Literature Search

**Figure 1.**
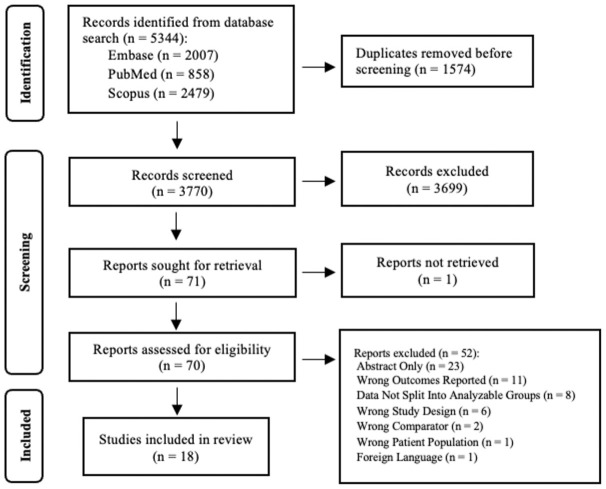
PRISMA (Preferred Reporting Items for Systematic Reviews and Meta-Analyses) flow diagram for systematic reviews and meta-analyses assessing osteoarthritis after the operative versus nonoperative treatment of anterior cruciate ligament injuries.

The initial search performed on June 2, 2025, yielded 5344 results, of which 1574 duplicates were removed. The remaining 3770 studies were screened, and 71 were assessed in a full-text review. Of these, 18 were included.^**^ The PRISMA flow diagram is shown in Figure 1.

### Study Quality

**Figure 2.**
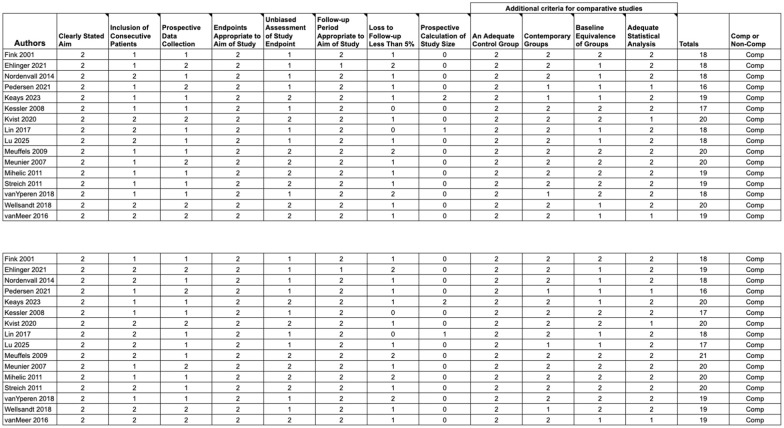
Methodological Index for Non-Randomized Studies (MINORS) scores for systematic reviews and meta-analyses assessing osteoarthritis after the operative versus nonoperative treatment of anterior cruciate ligament injuries.

**Figure 3.**
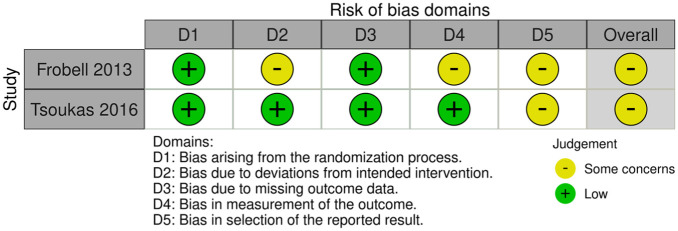
Cochrane RoB 2 (risk of bias tool) scores for systematic reviews and meta-analyses assessing osteoarthritis after the operative versus nonoperative treatment of anterior cruciate ligament injuries.

Among the 18 included studies, 1 (5.6%) was level 1 evidence,^16^ 9 (50.0%) were level 2,^††^ and 8 (44.4%) were level 3.^14,30,31,37,38,40,42,62^ The mean MINORS score was 18.8 for comparative studies, with no noncomparative studies being included. The Cochrane RoB 2 found that both RCTs had some concerns.^16,64^ The domains that most frequently raised concerns were deviations from intended interventions and selection of the reported result. In contrast, the randomization process and the completeness of outcome data were generally rated as low risk. The MINORS and Cochrane RoB 2 scores for individual studies are shown in Figures 2 and 3, respectively. Using the GRADE framework, the certainty of evidence was low for radiographic OA and TKA and very low for symptomatic OA, OA grade, secondary meniscal surgery, and adverse events (AppendixTable A2). Overall confidence in these estimates was limited by risk of bias, inconsistency, indirectness, and imprecision.

### Study and Patient Characteristics

This review included 18 studies with a total of 33,540 patients. Of these, 14,997 were in the operative group, and 18,179 were in the nonoperative group (Table 1). An additional 172 were in the early ACLR group, 50 were in the delayed ACLR group, and 142 were in the ACL repair group. Across the included studies, about 33% of patients were female. The mean age was 32.2 years (range, 19-59 years). The mean follow-up time was 147.6 months (or 12.3 years), and the mean loss to follow-up was 14.7%. Patient characteristics are presented in Table 1. Study characteristics are presented in AppendixTable A4. Associated injuries, baseline OA status, and concomitant procedures performed at the time of surgery were variably reported across studies and are summarized in AppendixTable A5.

**Table 1:** Patient Characteristics*^*a*^*

First Author (Year)	No. of Patients	No. of Knees	Female Sex, %	Age,*^*b*^* y	Follow-up,*^*c*^* mo	Loss to Follow-up, n (%)	Graft Type, %		
							BPTB	HT	ITB
Operative									
Frobell^16^ (2013)									
Early ACLR	59	59	20	26.6 ± 5.1	60	1 (2)	44	56	
Delayed ACLR	30	30	37	25.2 ± 4.5	60	0 (0)	44	56	
Fink^14^ (2001)	46	46	20	33.6 ± 8.0	132.2	26 (36)	1		
Ehlinger^11^ (2021)	228	228	59	54.8 ± 4.3	14.2 ± 3.8	0 (0)		86	
Nordenvall^46^ (2014)	11,940	11,940	35	29.93 ± 10.15	108 ± 60	18,979 (61)			
Pedersen^50^ (2021)									
Early ACLR	113	113	46	24.7 ± 8.7	60	54 (32)	22	78	
Delayed ACLR	20	20	30	24.4 ± 9.4	60	10 (33)	22	78	
Keays^30^ (2023)	56	56	30	38.7 ± 8.3	56 ± 108	6 (11)	52	48	
Kessler^31^ (2008)	60	60	38	30.7 (12.5-54.0)	133.2	NR	1		
Kvist^33^ (2020)									
ACL repair	64	64	27	24 ± 6	384-444	0 (0)			64
Delayed repair ≤2 y	27	27	26	24 ± 5	384-444	0 (0)			27
Delayed repair >2 y	9	9	56	19 ± 4	384-444	0 (0)			9
Lin^37^ (2017)	1374	1374	29	30.5 ± 11.3	216	NR			
Lu^38^ (2025)	974	974	42	26 (19-35)	161 (120-211)	663 (41)			
Meuffels^40^ (2009)	25	25	24	37.6 ± 6.2	120	0 (0)	1		
Meunier^41^ (2007)	42	42	25	22 (14-30)	126-228	2 (5)	NR		
Mihelic^42^ (2011)	36	36	19	25.3	204-240	0 (0)	1		
Streich^62^ (2011)	40	40	40	26.0 ± 6.3 (18-39)	185 ± 9	14 (18)	1		
Tsoukas^64^ (2016)	17	17	0	31 (20-36)	121	NR		1	
van Yperen^66^ (2018)	25	25	25	45.8 ± 6.4	254.6	0 (0)	1		
Wellsandt^69^ (2018)	83	83	33	33.6 ± 11.0	64.0 ± 9.6	39 (27)		1	
van Meer^65^ (2016)	93	93	NR	25.2 (21.4-32.6)	25.9	11 (7)	4	96	
Nonoperative									
Frobell^16^ (2013)	29	29	31	26.4 ± 4.9	60	0 (0)			
Fink^14^ (2001)	25	25	28	32.3 ± 9.9	140.0 ± 9.6	16 (39)			
Ehlinger^11^ (2021)	92	92	62	59.9 ± 6.6 (50.8-74.2)	18	0 (0)			
Nordenvall^46^ (2014)	9970	9970	36	29.93 ± 10.15	108 ± 60	23,725 (70)			
Pedersen^50^ (2021)	54	54	55	31.9 ± 10.9	60	11 (17)			
Keays^30^ (2023)	45	45	33	33.0 ± 6.2 (24-47)	132.0 ± 62.2	0 (0)			
Kessler^31^ (2008)	49	49	38	30.7 (12.5-54.0)	133.2	NR			
Kvist^33^ (2020)	53	53	32	25 ± 6	384-444	0 (0)			
Lin^37^ (2017)	7395	7395	46	40.3 ± 16.0	216	NR			
Lu^38^ (2025)	220	220	45	38.5 (32-45)	200 (151-251)	140 (38)			
Meuffels^40^ (2009)	25	25	24	37.8 ± 6.8	120	0 (0)			
Meunier^41^ (2007)	52	52	38	21 (14-30)	126-228	4 (7)			
Mihelic^42^ (2011)	18	18	19	25.5	204-240	0 (0)			
Streich^62^ (2011)	40	40	40	24.0 ± 6.5 (17-38)	183 ± 8	14 (18)			
Tsoukas^64^ (2016)	15	15	0	33 (25-39)	121	NR			
van Yperen^66^ (2018)	25	25	25	45.8 ± 6.4	289.4	0 (0)			
Wellsandt^69^ (2018)	22	22	55	33.6 ± 11.0	61.2 ± 7.2	NR			
van Meer^65^ (2016)	50	50	0	25.2 (21.4-32.6)	25.9	NR			

a ACL, anterior cruciate ligament; ACLR, anterior cruciate ligament reconstruction; BPTB, bone–patellar tendon–bone; HT, hamstring tendon; ITB, iliotibial band; NR, not reported.

b Data are shown as mean ± SD, mean (range), or mean ± SD (range).

c Data are shown as mean, mean ± SD, range, or mean (range).

### Radiographic OA

**Figure 4.**
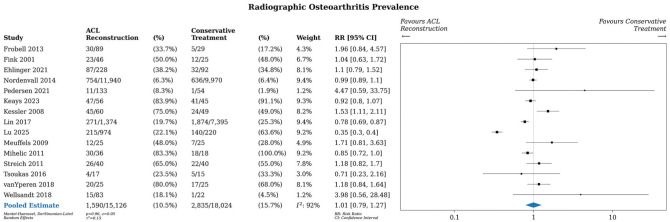
Forest plot (random effects) demonstrating a meta-analysis of osteoarthritis prevalence in all studies comparing the anterior cruciate ligament reconstruction group and nonoperative group with associated risk ratios, heterogeneity calculations, and 95% confidence intervals. The pooled estimate is presented for completeness; however, because of substantial heterogeneity, this estimate should not be considered clinically interpretable.

**Figure 5.**
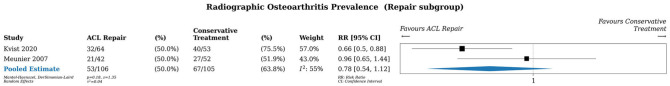
Forest plot (random effects) demonstrating a meta-analysis of osteoarthritis prevalence in all studies comparing the anterior cruciate ligament repair group and nonoperative group with associated risk ratios, heterogeneity calculations, and 95% confidence intervals.

All 18 of the included studies reported radiographic OA, which can be seen in Table 2. A meta-analysis of 15 studies reporting the prevalence of OA in the ACLR versus nonoperative group had high heterogeneity and found no statistically significant difference in rates (RR, 1.01 [95% CI, 0.79-1.27]; *I*^2^ = 92%; *P* = .96) (Figure 4). A meta-analysis of 2 studies assessing OA prevalence in the ACL repair versus nonoperative group also found no statistically significant difference, with moderate heterogeneity (RR, 0.78 [95% CI, 0.54-1.12]; *I*^2^ = 55%; *P* = .18) (Figure 5). This analysis should be considered exploratory, given the limited number of included studies.

**Table 2:** OA Outcomes*^*a*^*

First Author (Year)	Radiographic OA	Symptomatic OA	Fairbank				KL				
			Grade 0	Grade 1	Grade 2	Grade 3	Grade 0	Grade 1	Grade 2	Grade 3	Grade 4
Operative											
Frobell^16^ (2013)											
Early ACLR	23 (39)	NR									
Delayed ACLR	7 (23)	NR									
Fink^14^ (2001)	23 (50)	NR	10 (22)	13 (28)	17 (37)	6 (13)					
Ehlinger^11^ (2021)	87 (38)	NR									
Nordenvall^46^ (2014)	745 (6)	NR									
Pedersen^50^ (2021)											
Early ACLR	8 (7)	NR						40 (35)	24 (21)		
Delayed ACLR	3 (15)	NR						5 (25)	4 (20)		
Keays^30^ (2023)	47 (84)	NR									
Kessler^31^ (2008)	45 (75)	NR					27 (45)	6 (10)	25 (42)	2 (3)	
Kvist^33^ (2020)											
ACL repair	20 (31)	28 (44)									
Delayed repair ≤2 y	8 (30)	14 (52)									
Delayed repair >2 y	2 (22)	6 (67)									
Lin^37^ (2017)	271 (20)	NR									
Lu^38^ (2025)	215 (22)	NR									
Meuffels^40^ (2009)	12 (48)	NR					4 (16)	9 (36)	9 (36)	3 (12)	
Meunier^41^ (2007)	21 (50)	NR	20 (48)	9 (21)	10 (24)	2 (5)					
Mihelic^42^ (2011)	30 (83)	NR									
Streich^62^ (2011)	26 (65)	NR									
Tsoukas^64^ (2016)	4 (24)	NR									
van Yperen^66^ (2018)	20 (80)	NR					1 (4)	4 (16)	16 (64)	3 (12)	
Wellsandt^69^ (2018)	15 (18)	NR									
van Meer^65^ (2016)	NR	NR									
Nonoperative											
Frobell^16^ (2013)	5 (17)	NR									
Fink^14^ (2001)	12 (48)	NR	4 (16)	9 (36)	3 (12)	9 (36)					
Ehlinger^11^ (2021)	32 (35)	NR									
Nordenvall^46^ (2014)	636 (6)	NR									
Pedersen^50^ (2021)	1 (2)	NR						17 (32)	10 (19)		
Keays^30^ (2023)	41 (91)	NR									
Kessler^31^ (2008)	24 (49)	NR					30 (61)	7 (14)	10 (20)	2 (4)	
Kvist^33^ (2020)	16 (30)	28 (53)									
Lin^37^ (2017)	1874 (25)	NR									
Lu^38^ (2025)	140 (64)	NR									
Meuffels^40^ (2009)	7 (28)	NR					8 (32)	10 (40)	4 (16)	3 (12)	
Meunier^41^ (2007)	27 (52)	NR	25 (48)	11 (21)	10 (19)	6 (12)					
Mihelic^42^ (2011)	18 (100)	NR									
Streich^62^ (2011)	22 (55)	NR									
Tsoukas^64^ (2016)	5 (33)	NR									
van Yperen^66^ (2018)	17 (68)	NR					3 (12)	5 (20)	12 (48)	4 (16)	1 (4)
Wellsandt^69^ (2018)	1 (5)	NR									
van Meer^65^ (2016)	NR	NR									

a Data are shown as n (%). ACL, anterior cruciate ligament; ACLR, anterior cruciate ligament reconstruction; KL, Kellgren-Lawrence; OA, osteoarthritis; NR, not reported.

**Figure 6.**
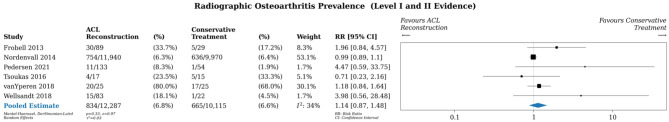
Forest plot (random effects) demonstrating a meta-analysis of osteoarthritis prevalence in level 1 and 2 studies comparing the anterior cruciate ligament reconstruction group and nonoperative group with associated risk ratios, heterogeneity calculations, and 95% confidence intervals.

A meta-analysis of 6 studies, considered level 1 or 2 evidence, exhibited low heterogeneity; therefore, the results were pooled. This analysis demonstrated no statistically significant difference in the prevalence of OA in either group (RR, 1.14 [95% CI, 0.87-1.48]; *I*^2^ = 34%; *P* = .33) (Figure 6).

However, 3 of the studies found significantly lower rates of radiographic OA in the ACLR group than the nonoperative group.^37,38,42^ One study reported a lower cumulative incidence of OA in the ACLR group compared to the nonoperative group (19.7% vs 25.3%, respectively; *P* < .001).^37^ Another reported posttraumatic radiographic OA at follow-up in 22% of the ACLR group compared to 64% of the nonoperative group (*P* < .001).^38^ Finally, one study found 16.5% of the ACLR group to have severe OA compared to 56.0% of the nonoperative group (*P* < .05).^42^

There were 5 studies that specified the anatomic regions of knee OA, including tibiofemoral, patellofemoral, anterior, posterior, medial, and lateral.^11,16,30,33,64^ One study found less medial OA in the nonoperative group (*P* = .021) and less patellofemoral OA in the ACLR group (*P* = .001).^11^ One study did not find any statistically significant difference between the ACLR and nonoperative groups but found more patellofemoral OA in knees reconstructed using patellar tendons compared to hamstring tendons (*P* = .001).^16^ Additionally, one study found that the ACL repair group had a lower prevalence of tibiofemoral OA compared to the nonoperative group (50% vs 75%, respectively; *P* = .005).^33^ No other studies found any significant difference in OA between the operative and nonoperative groups.

### Symptomatic OA

One study reported symptomatic OA and found no significant difference between the ACL repair group, nonoperative group, and delayed ACL repair group.^33^

### OA Grade

Overall, 3 studies used the IKDC radiographic grading system.^42,62,64^ There were 2 studies that found higher OA rates in the operative group,^31,62^ with one showing statistical significance.^31^ One study found statistically significantly higher OA rates in the nonoperative group.^42^ Additionally, 7 studies used the KL scale.^16,31,33,40,50,66,69^ Based on KL grades between groups, one study found the nonoperative group to have a lower risk of developing OA than the ACLR group (24% vs 45%, respectively; *P* = .03).^31^ All the other studies showed no significant difference in KL grades between groups. One study used the Fairbank classification of OA and found no significant difference between the ACLR and nonoperative groups.^14^ The Ahlbäck and Fairbank classifications were used by another study, with no significant difference between the ACL repair and nonoperative groups.^41^

### Secondary Meniscal Surgery

**Figure 7.**
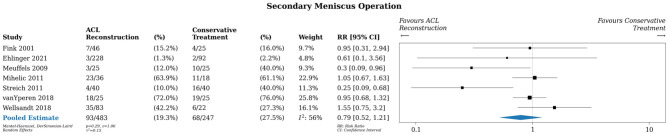
Forest plot (random effects) demonstrating a meta-analysis of secondary meniscal surgery in the anterior cruciate ligament reconstruction group and nonoperative group with associated risk ratios, heterogeneity calculations, and 95% confidence intervals.

A total of 677 patients (30.3%) from 12 studies required secondary meniscal surgery, including meniscal repair or meniscectomy (Table 3).^‡‡^ A meta-analysis of 7 studies reporting secondary meniscal surgery found no statistically significant difference between groups (RR, 0.79 [95% CI, 0.52-1.21]; *I*^2^ = 56%; *P* = .29) (Figure 7). One study reported a lower secondary meniscal surgery rate in the ACLR group compared to the nonoperative group (68% [n = 17] vs 84% [n = 21], respectively; *P* = .024).^40^ Another study also found a lower incidence of secondary partial meniscectomy in the ACLR group than the nonoperative group (10% [n = 4] vs 40% [n = 16], respectively; *P* = .028).^62^ No other studies found a significant difference in secondary meniscal surgery between groups.

**Table 3:** Secondary Meniscal Surgery and TKA*^*a*^*

First Author (Year)	Secondary Meniscal Surgery		TKA
	Meniscal Repair	Meniscectomy	
Operative			
Frobell^16^ (2013)			
Early ACLR	29 (49)		NR
Delayed ACLR	26 (87)		NR
Fink^14^ (2001)	7 (15)		NR
Ehlinger^11^ (2021)	3 (1)		NR
Nordenvall^46^ (2014)	NR		444 (4)
Pedersen^50^ (2021)			
Early ACLR	NR		NR
Delayed ACLR	NR		NR
Keays^30^ (2023)	NR		NR
Kessler^31^ (2008)	NR		NR
Kvist^33^ (2020)			
ACL repair	21 (33)		5 (8)
Delayed repair ≤2 y	10 (37)		3 (11)
Delayed repair >2 y	3 (33)		0 (0)
Lin^37^ (2017)	479 (35)		5 (0.4)
Lu^38^ (2025)	NR		25 (3)
Meuffels^40^ (2009)	17 (68)		NR
Meunier^41^ (2007)	5 (12)		NR
Mihelic^42^ (2011)	23 (64)		NR
Streich^62^ (2011)		4 (10)	NR
Tsoukas^64^ (2016)	NR		NR
van Yperen^66^ (2018)		4 (16)	1 (4)
Wellsandt^69^ (2018)	35 (42)		NR
van Meer^65^ (2016)	8 (9)	3 (3)	NR
Nonoperative			
Frobell^16^ (2013)	23 (79)		NR
Fink^14^ (2001)	4 (16)		NR
Ehlinger^11^ (2021)	2 (2)		NR
Nordenvall^46^ (2014)	NR		444 (5)
Pedersen^50^ (2021)	NR		NR
Keays^30^ (2023)	NR		NR
Kessler^31^ (2008)	NR		NR
Kvist^33^ (2020)	0 (0)		3 (6)
Lin^37^ (2017)	259 (4)		154 (2)
Lu^38^ (2025)	NR		50 (23)
Meuffels^40^ (2009)	21 (84)		NR
Meunier^41^ (2007)	18 (35)		NR
Mihelic^42^ (2011)	11 (61)		NR
Streich^62^ (2011)		16 (40)	NR
Tsoukas^64^ (2016)	NR		0 (0)
van Yperen^66^ (2018)		10 (40)	0 (0)
Wellsandt^69^ (2018)	6 (27)		NR
van Meer^65^ (2016)		2 (4)	NR

a Data are shown as n (%). ACL, anterior cruciate ligament; ACLR, anterior cruciate ligament reconstruction; NR, not reported; TKA, total knee arthroplasty.

### Total Knee Arthroplasty

**Figure 8.**
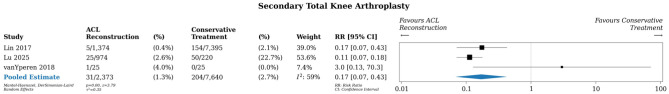
Forest plot (random effects) demonstrating a meta-analysis of total knee arthroplasty in the anterior cruciate ligament reconstruction group and nonoperative group with associated risk ratios, heterogeneity calculations, and 95% confidence intervals.

A total of 1134 patients (3.4%) from 5 studies required TKA.^33,37,38,46,66^ A meta-analysis of 3 studies reporting TKA found a statistically significant reduction in the need for TKA in the ACLR group (RR, 0.17 [95% CI, 0.07-0.43]; *I*^2^ = 59%; *P* = <.001) (Figure 8). One study reported that fewer patients from the ACLR group required TKA compared to the nonoperative group (n = 25 [3%] vs 50 [23%], respectively; *P* < .001).^38^ No other studies found a significant difference in TKA between groups.

**Figure A1.**
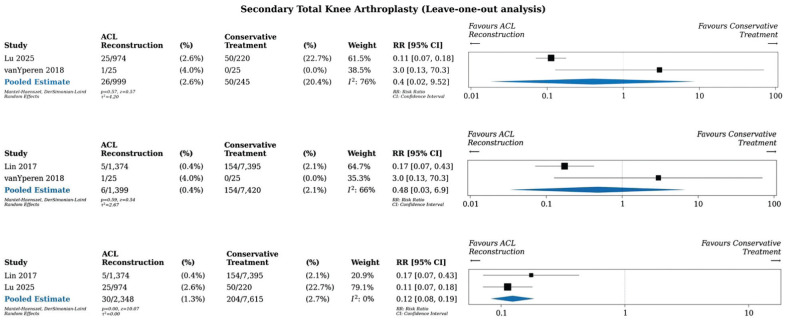
Forest plots (random effects) demonstrating 3 leave-one-out meta-analyses of total knee arthroplasty in the anterior cruciate ligament reconstruction group and nonoperative group with associated risk ratios, heterogeneity calculations, and 95% confidence intervals.

Leave-one-out sensitivity analysis demonstrated that the direction of effect remained consistent across all iterations; however, statistical significance was sensitive to the removal of individual large studies. The exclusion of either of 2 studies^37,38^ resulted in the loss of significance, while the exclusion of a smaller study^66^ strengthened the observed effect (RR, 0.12 [95% CI, 0.08-0.19]; *I*^2^ = 0%; *P* < .001) (Appendix Figure A1). These findings suggest that the observed reduction in the need for TKA may be influenced by study weighting and should be interpreted with caution.

### Adverse Events

A total of 8 patients who had undergone ACLR had a graft rupture (partial or full) or residual laxity during the follow-up period after the initial intervention.^11,14,16^ One study reported 3 graft ruptures in the early ACLR group and 1 graft rupture in the delayed ACLR group,^16^ while another reported 1 partial graft rupture in the ACLR group.^14^ Residual laxity or graft failure was reported in one study in 1 patient in the nonoperative group and 2 patients in the ACLR group.^11^

## Discussion

The primary finding of this study is that the surgical management of ACL injuries did not reduce the prevalence of radiographic knee OA compared to nonoperative care. This review did, however, identify a statistically significant reduction in the need for TKA in the ACLR group.

While operative management is effective at restoring knee stability and reducing anterior laxity compared with nonoperative management,^40,66^ it is unclear whether there is a decreased risk of developing OA. Several studies have reported lower OA rates after ACLR; however, pooled data of high-quality studies do not support this conclusion. A 2021 meta-analysis found no difference in OA prevalence between the ACLR and nonoperative groups, although it reported improved functional outcomes and lower rates of secondary meniscectomy in the ACLR group.^7^ Further, a systematic review and meta-analysis with >20-year follow-up demonstrated a high prevalence of knee OA after ACLR, especially in patients with concomitant chondral or meniscal injuries, older age, and delayed surgery.^19^ Another study with >10-year follow-up reported a higher OA prevalence but fewer cases of secondary meniscectomy after ACLR.^36^ These findings are in contrast with another study that reported a 3.89-fold increased RR of OA at 10 years after an ACL injury, regardless of treatment, but found nonoperative patients at a greater risk.^1^ Moreover, a meta-analysis of 3 RCTs found a statistically significant increase in OA in patients after ACLR compared with controls.^13^ The inconsistent findings are likely driven by the heterogeneity in OA grading scales, surgical techniques, rehabilitation protocols, and follow-up durations. In contrast to previous studies, this review included >33,000 patients; applied standardized radiographic criteria; and evaluated OA, secondary meniscal surgery, and TKA as distinct outcomes. We believe that this approach provides a more comprehensive assessment of long-term joint health.

The development of OA after ACL injuries is multifactorial. The intact ACL prevents anterior tibial translation and internal tibial rotation.^10^ When the ACL is ruptured, the knee's rotary axis is shifted, altering the flexion arc of the medial femoral condyle.^2,29^ In an ACL-deficient knee, the posterior horn of the medial meniscus becomes the primary restraint to anterior tibial translation.^9,18^ These altered kinematics increase contact forces and chondral damage, in addition to predisposing patients to secondary medial meniscal injuries. This is particularly relevant, as 71% of ACL-deficient knees sustain medial meniscal tears, with 81.8% involving the posterior horn.^17^ Although ACLR has not reliably been shown to prevent long-term OA, it is hypothesized that preserving meniscal integrity indirectly reduces the risk.^31,58^ A meniscal injury was associated with a 15-fold increase in the odds of TKA for OA in one study,^32^ backed by another study that reported that a 10% reduction in meniscal contact area can increase peak joint contact stress by 65%.^4^ Meniscectomy has also been shown to be a strong predictor of posttraumatic OA.^55^ This meta-analysis did not show a statistically significant difference in secondary meniscal surgery rates between groups. This outcome should be interpreted cautiously, as it represents a surrogate for cumulative meniscal abnormalities and is inconsistently reported. Additionally, 13 included studies noted concomitant meniscal interventions (repair or partial meniscectomy) performed during index surgery, which may have reduced the need for later operative procedures.

Posttraumatic OA after an ACL injury may also result in part from bone bruising sustained during the “pivot-shift” mechanism of the injury^48,52^ and the associated inflammatory cascade.^5,27^ The surgical management of ACL injuries may contribute to OA through altered knee kinematics and procedure-induced inflammation.^26^ Variations in surgical technique and graft selection might affect long-term joint degeneration^51^; however, evidence for this is inconclusive.^67^ These factors support the view that ACLR alone is not sufficient to prevent posttraumatic OA.^8^ Ultimately, concomitant lesions may play a more significant role in long-term joint health than ACL status alone. Variations in the presence, severity, and management of these associated lesions across studies likely contribute to the heterogeneity observed and may partially explain the lack of a consistent protective effect of surgical reconstruction.

It is also important to consider rehabilitation and preoperative physical therapy when evaluating the development of long-term OA. Every 1% increase in the quadriceps’ limb symmetry index has been shown to reduce the odds of clinical OA at 5 years by 4%, while the ACL's surgical status itself was not found to be protective in the same study.^3^ Another study demonstrated that quadriceps latency before ACL surgery was the only predictor of radiographic OA at 6 years.^28^ These findings highlight the importance of preoperative physical therapy.

The distinction between radiographic and symptomatic OA is an important consideration. One study included in this review separated these outcomes.^33^ Another study was a long-term case series, demonstrating that while 75% of patients had radiographic OA at >30 years after an ACL injury, only 38% were symptomatic.^21^ This distinction is clinically relevant, as radiographic OA alone may overestimate the functional burden. This meta-analysis found a low incidence of TKA after ACLR (3.2%). Registry data have similarly demonstrated a 15-year risk of only 1.1%, with cartilage injuries and revision surgery identified as risk factors.^68^ These authors also found that lower patient-reported knee function at 2 years after ACLR independently predicted a higher risk of TKA.^68^ The discordance between similar radiographic OA rates and lower TKA rates after ACLR may reflect differences in symptomatic progression rather than structural degeneration alone. ACLR may improve joint stability and functional outcomes, potentially delaying the progression to symptomatic OA, despite similar radiographic changes. These interpretations remain speculative and require further investigation.

The strengths of this review include the large pooled sample size, long-term follow-up durations, and rigorous methodology. We utilized standardized OA definitions, performed a detailed risk of bias assessment, and conducted sensitivity analyses excluding highly heterogeneous studies. The findings of this study demonstrate that ACLR may reduce the need for TKA but does not reliably prevent the development of radiographic OA compared to nonoperative management. Joint preservation after ACL injuries is multifactorial, including meniscal preservation, knee biomechanics, and optimized rehabilitation. Future research comparing surgical techniques, cartilage restoration procedures, and structured prehabilitation/rehabilitation protocols is warranted to better understand the long-term relationship between ACL injuries and varying management strategies. The development of knee OA should be monitored both radiographically and clinically, with an individualized approach to the treatment of ACL injuries.

### Limitations

The heterogeneity observed across studies is likely multifactorial, including variations in OA assessments, surgical techniques and graft choice, timing of reconstruction, rehabilitation protocols, severity of the baseline injury, and follow-up durations. Because of inconsistent reporting of these variables across studies, a formal meta-regression was not feasible. The included studies span multiple decades, encompassing a substantial evolution in the management of ACL injuries. Earlier studies often reflect nonanatomic ACLR techniques and less standardized rehabilitation, whereas more recent studies incorporate anatomic reconstruction principles and structured rehabilitation protocols. These temporal differences may also influence long-term joint outcomes and contribute to the heterogeneity observed across studies. Although radiographic OA definitions were standardized across classification systems, the KL, IKDC, and Fairbank grading systems are not fully interchangeable. Additionally, collapsing compartment-specific OA into a single OA outcome may obscure differences between tibiofemoral and patellofemoral disease. Inconsistent reporting of associated injuries, concomitant procedures, and baseline OA status across studies further limits the ability to isolate the independent effect of treatment strategy on long-term outcomes. A lot of the included studies were level 3, also increasing the potential risk of bias. Only 10 level 1 or 2 studies were available in the literature, limiting the power of high-quality pooled estimates. Further, reporting of secondary meniscal injuries was inconsistent, restricting further subgroup analyses. Many included studies had a high loss to follow-up, increasing the risk of attrition bias and limiting the reliability of long-term outcomes. The restriction to studies in English or with available English translations may introduce language bias.

Confounding by indication is an important limitation of the included observational studies. Patients undergoing ACLR are often younger, are more active, and have higher functional demands, while nonoperative cohorts may include lower demand patients. These baseline differences may influence both OA progression and the likelihood of undergoing TKA. The observed reduction in TKA rates after ACLR should be interpreted as an association rather than a causal effect, as treatment selection and patient characteristics may influence this outcome.

## Conclusion

The operative management of ACL injuries did not decrease the long-term risk of developing radiographic OA compared with nonoperative management. ACLR was associated with a lower rate of TKA.

## Footnotes

** References 11,14,16,30,31,33,37,38,40,41,42,46,50,62,64,65,66,69.

†† References 11,33,41,46,50,64,65,66,69.

‡‡ References 11,14,16,33,37,40,41,42,62,65,66,69.

## Appendix

**Table A1:** Search Strategy

PubMed (n = 858)	Embase (n = 2007)	Scopus (n = 2479)
1. “Anterior Cruciate Ligament” [MeSH Terms]	1. ‘Anterior Cruciate Ligament’/exp OR acl:ti,ab	TITLE-ABS-KEY(“anterior cruciate ligament injury” OR “ACL tear” OR “ACL rupture” OR “ACL injury” OR “ACL reconstruction”)
2. “ACL” [Title/Abstract]	2. ‘Anterior Cruciate Ligament Injury’:ti,ab	)AND(
3. “Anterior Cruciate Ligament Injury” [Title/Abstract]	3. 1 OR 2	TITLE-ABS-KEY(“osteoarthritis” OR “post-traumatic osteoarthritis” OR “joint degeneration”)
4. 1 OR 2 OR 3	4. ‘Osteoarthritis’/exp	)AND(
5. “Osteoarthritis” [MeSH Terms]	5. ‘Post-traumatic Osteoarthrtis’:ti,ab	TITLE-ABS-KEY(“operative” OR “surgical” OR “reconstruction” OR “nonoperative” OR “non-operative” OR “conservative treatment” OR “rehabilitation”)
6. “Post-traumatic Osteoarthritis” [Title/Abstract]	6. ‘Joint Degeneration’:ti,ab	)AND(
7. “Joint Degeneration” [Title/Abstract]	7. 4 OR 5 OR 6	TITLE-ABS-KEY(“outcomes” OR “incidence” OR “risk” OR “follow-up” OR “radiographic” OR “progression”)
8. 5 OR 6 OR 7	8. ‘Anterior Cruciate Ligament Reconstruction’/exp	)AND(
9. “Reconstruction” [Title/Abstract]	9. ‘Reconstruction’:ti,ab	LIMIT-TO(LANGUAGE, “English”)
10. “Surgical” [Title/Abstract]	10. ‘Nonoperative Treatment’:ti,ab	
11. “Nonoperative” [Title/Abstract]	11. ‘Conservative’:ti,ab	
12. “Conservative Treatment” [Title/Abstract]	12. ‘Rehabilitation’:ti,ab	
13. 10 OR 11 OR 12	13. 8 OR 9 OR 10 OR 11 OR 12	
14. 4 AND 8 AND 13	14. 3 AND 7 AND 13	

**Table A2:** Certainty of Evidence Using GRADE Framework*^*a*^*

No. of Studies	Study Design	Risk of Bias	Inconsistency	Indirectness	Imprecision	Other Considerations	ACLR, n (%)	Nonoperative Management, n (%)	RR (95% CI)	Absolute Risk (95% CI)	Certainty	Importance
Radiographic OA												
15	Nonrandomized studies*^*b*^*	Serious*^*c*^*	Serious*^*d*^*	Not serious	Not serious	None	1590/15,126 (10.5)	2835/18,024 (15.7)	1.01(0.79-1.27)	2 more per 1000 (from 33 fewer to 42 more)	⊕⊕◯◯Low	Critical
Symptomatic OA												
1	Nonrandomized studies	Serious*^*e*^*	Not serious	Not serious	Serious*^*f*^*	None	One cohort study reported symptomatic OA and found no significant difference between the ACL repair, nonoperative management, and delayed repair groups.				⊕◯◯◯Very low	Critical
OA grade												
9	Nonrandomized studies	Serious*^*g*^*	Serious*^*h*^*	Serious*^*i*^*	Serious*^*j*^*	None	A total of 9 studies using various OA grading systems (IKDC, KL, Fairbank, and Ahlbäck) reported variable findings, with some studies demonstrating higher OA severity in the operative group, one study showing higher severity in the nonoperative group, and several studies reporting no significant difference.				⊕◯◯◯Very low	Critical
Secondary meniscal surgery												
7	Nonrandomized studies	Serious*^*g*^*	Serious*^*k*^*	Not serious	Serious*^*l*^*	None	93/483 (19.3)	68/247 (27.5)	0.79 (0.52-1.21)	58 fewer per 1000(from 132 fewer to 58 more)	⊕◯◯◯Very low	Critical
TKA												
3	Nonrandomized studies*^*m*^*	Serious*^*g*^*	Not serious*^*n*^*	Not serious	Not serious	Strong association	31/2373 (1.3)	204/7640 (2.7)	0.17(0.07-0.43)	22 fewer per 1000(from 25 fewer to 15 fewer)	⊕⊕◯◯Low	Critical
Adverse events												
3	Nonrandomized studies*^*o*^*	Serious*^*g*^*	Not serious	Serious*^*p*^*	Very serious*^*q*^*	None	Adverse events were infrequently and variably reported across 3 studies, including graft ruptures and residual laxity, with no clear difference between the operative and nonoperative groups.				⊕◯◯◯Very low	Critical

a ACL, anterior cruciate ligament; ACLR, anterior cruciate ligament reconstruction; GRADE, Grading of Recommendations Assessment, Development and Evaluation; IKDC, International Knee Documentation Committee; KL, Kellgren-Lawrence; OA, osteoarthritis; RR, risk ratio; TKA, total knee arthroplasty.

b These included 2 randomized studies and 13 cohort studies.

c Most included studies were observational cohort studies with an inherent risk of bias, and although a small number of randomized trials were included, sensitivity analyses restricted to higher level evidence did not change the overall findings.

d There was high heterogeneity in the primary analysis of ACLR versus nonoperative management (*I*^2^ = 92%). Although heterogeneity was lower in sensitivity analyses restricted to higher level evidence (*I*^2^ = 34%), effect estimates remained variable across analyses. Additionally, subgroup analysis of ACL repair versus nonoperative management demonstrated a differing direction of effect, further supporting serious inconsistency.

e Evidence was derived from a single observational cohort study with an inherent risk of bias.

f Evidence was limited to a single study with a small sample size and no statistically significant difference, resulting in uncertainty around the effect estimate.

g Most included studies were observational cohort studies with an inherent risk of bias.

h Findings were variable across studies, with some demonstrating higher OA severity in the operative group, one showing higher severity in the nonoperative group, and several reporting no significant difference.

i Multiple different OA grading systems were used across studies, limiting the comparability and direct interpretation of results.

j Data were not pooled, and effect estimates were inconsistently reported, limiting precision and preventing quantitative synthesis.

k Moderate heterogeneity was observed across studies (*I*^2^ = 56%), with some studies demonstrating reduced reoperation rates in the operative group and others showing no difference.

l The pooled estimate was imprecise, with a CI crossing the null effect (RR, 0.79 [95% CI, 0.52-1.21]), and relatively small sample sizes contributed to uncertainty.

m There were 5 studies that reported this outcome, with 3 included in the pooled analysis.

n Moderate heterogeneity was observed (*I*^2^ = 59%), with variations in effect estimates across studies.

o These included 1 randomized study and 2 cohort studies.

p Adverse events were variably defined and reported (eg, graft rupture, residual laxity), limiting comparability across studies.

q Very few events were reported across studies, resulting in substantial uncertainty and an inability to determine the effect estimate.

**Table A3:** OA Definitions Corresponding to Standardized Definitions*^*a*^*

First Author (Year)	OA Classification System Used	Standardized Definition
Ehlinger^11^ (2021)	Mild to moderate: ≤50% narrowing or joint space of 2-4 mm Severe: >50% narrowing or joint space <2 mm	For studies reporting descriptively, all categories were incorporated into the prevalence estimate
Fink^14^ (2001)	Fairbank	Fairbank: moderate to severe
Frobell^16^ (2013)	KL	KL grade ≥2
Keays^30^ (2023)	Similar to KL	KL grade ≥2
Kessler^31^ (2008)	KL	KL grade ≥2
Kvist^33^ (2020)	KL	KL grade ≥2
Lin^37^ (2017)	OA diagnosis via national registry coding	For studies reporting descriptively, all categories were incorporated into the prevalence estimate
Lu^38^ (2025)	Moderate to severe degenerative changes on radiography	For studies reporting descriptively, all categories were incorporated into the prevalence estimate
Meuffels^40^ (2009)	KL	KL grade ≥2
Meunier^41^ (2007)	Fairbank	Fairbank: moderate to severe
Mihelic^42^ (2011)	IKDC	IKDC grades B-D
Nordenvall^46^ (2014)	OA diagnosis via national registry coding	For studies reporting descriptively, all categories were incorporated into the prevalence estimate
Pedersen^50^ (2021)	KL	KL grade ≥2
Streich^62^ (2011)	IKDC	IKDC grades B-D
Tsoukas^64^ (2016)	IKDC	IKDC grades B-D
van Meer^65^ (2016)	MOAKS	For studies reporting descriptively, all categories were incorporated into the prevalence estimate
van Yperen^66^ (2018)	KL	KL grade ≥2
Wellsandt^69^ (2018)	KL	KL grade ≥2

a IKDC, International Knee Documentation Committee; KL, Kellgren-Lawrence; MOAKS, MRI Osteoarthritis Knee Score; OA, osteoarthritis.

**Table A4:** Study Characteristics*^*a*^*

First Author (Year)	Study Period	Study Design	MINORS Score	LOE	Group 1	Group 2	Group 3
Frobell^16^ (2013)	2002-2006	RCT	NA	1	ACLR	Delayed ACLR	Rehabilitation only
Fink^14^ (2001)	1981-1983	Retrospective cohort	18	3	ACLR	Rehabilitation only	NA
Ehlinger^11^ (2021)	2016-2018	Prospective comparative series	18.5	2	ACLR	Rehabilitation only	NA
Nordenvall^46^ (2014)	1987-2009	Population cohort	18	2	ACLR	Rehabilitation only	NA
Pedersen^50^ (2021)	2006-2012	Prospective cohort	16	2	Early ACLR	Delayed ACLR	Rehabilitation only
Keays^30^ (2023)	NR	Prospective and retrospective cohort	19.5	3	ACLR	Rehabilitation only	NA
Kessler^31^ (2008)	1989-1997	Retrospective cohort	17	3	ACLR	Rehabilitation only	NA
Kvist^33^ (2020)	1980-1985	Prospective cohort	20	2	ACL repair	Delayed repair	Rehabilitation only
Lin^37^ (2017)	1996-2013	Retrospective cohort	18	3	ACLR	No ACLR	NA
Lu^38^ (2025)	1990-2016	Cohort	17.5	3	ACLR	Rehabilitation only	NA
Meuffels^40^ (2009)	1994-1996	Matched-pair cohort	20.5	3	ACLR	Rehabilitation only	NA
Meunier^41^ (2007)	1980-1983	Prospective cohort	20	2	ACL repair	Rehabilitation only	NA
Mihelic^42^ (2011)	1989-1991	Retrospective cohort	19.5	3	ACLR	Rehabilitation only	NA
Streich^62^ (2011)	1990-1992	Retrospective case series	19.5	3	ACLR	Rehabilitation only	NA
Tsoukas^64^ (2016)	NR	Prospective randomized	NA	2	ACLR	Rehabilitation only	NA
van Yperen^66^ (2018)	1992-1996	Cohort	18.5	2	ACLR	Rehabilitation only	NA
Wellsandt^69^ (2018)	2005-2011	Cohort	19.5	2	ACLR	Rehabilitation only	NA
van Meer^65^ (2016)	2009-2010	Cohort	19	2	ACLR	Rehabilitation only	NA

a ACL, anterior cruciate ligament; ACLR, anterior cruciate ligament reconstruction; LOE, level of evidence; MINORS, Methodological Index for Non-Randomized Studies; NA, not applicable; NR, not reported; RCT, randomized controlled trial.

**Table A5:** Associated Injuries, Baseline OA Status, and Concomitant Procedures*^*a*^*

First Author (Year)	Associated Injuries	Baseline OA Status	Concomitant Procedures
Frobell^16^ (2013)	NR	NR	Partial meniscectomy or meniscal repair depending on meniscal status: NR
Fink^14^ (2001)	ACLR: medial meniscal injury, 11 (15.3%); lateral meniscal injury, 12 (16.7%); MCL injury, 7 (9.7%) Rehabilitation: medial meniscal injury, 6 (14.6%); lateral meniscal injury, 5 (12.2%); MCL injury, 4 (9.8%)	ACLR: preoperative Fairbank grade 0, 59 (82.6%); preoperative Fairbank grade 1, 13 (17.4%) Rehabilitation: preoperative Fairbank grade 0, 27 (65.2%); preoperative Fairbank grade 1, 14 (34.8%)	Partial meniscectomy: NR
Ehlinger^11^ (2021)	ACLR: medial meniscal injury, 83 (36%); lateral meniscal injury, 25 (11%); medial + lateral meniscal injury, 46 (20%); medial chondral injury, 30 (13%); lateral chondral injury, 4 (2%); patellar chondral injury, 17 (7%); mixed chondral injury, 122 (54%)	ACLR: mild medial OA, 37 (16%); moderate medial OA, 13 (6%); mild lateral OA, 9 (4%); moderate lateral OA, 3 (1%); mild patellofemoral OA, 18 (8%); moderate patellofemoral OA, 4 (2%) Rehabilitation: mild medial OA, 8 (9%); moderate medial OA, 2 (2%); mild lateral OA, 4 (4%); moderate lateral OA, 1 (1%); mild patellofemoral OA, 19 (21%); moderate patellofemoral OA, 1 (1%)	Anterolateral reconstruction: 37 (16%) Medial meniscal surgery: left in situ, 17 (7%); resected, 77 (34%); sutured, 25 (11%); prior resection sequela, 10 (4%) Lateral meniscal surgery: left in situ, 29 (13%); resected, 24 (11%); sutured, 16 (7%); prior resection sequela, 2 (1%)
Nordenvall^46^ (2014)	ACLR: meniscal injury, 5287 (44%) Rehabilitation: meniscal injury, 3102 (31%)	NR	NR
Pedersen^50^ (2021)	Early ACLR: medial meniscal injury, 45 (27%); lateral meniscal injury, 34 (20%); cartilage injury, 12 (7%); MCL injury (grade 1-2), 39 (23%); LCL injury (grade 1-2), 3 (2%) Delayed ACLR: medial meniscal injury, 8 (27%); lateral meniscal injury, 7 (23%); cartilage injury, 5 (17%); MCL injury, 6 (20%); LCL injury, 1 (3%) Rehabilitation: medial meniscal injury, 7 (11%); lateral meniscal injury, 6 (9%); cartilage injury, 5 (8%); MCL injury, 11 (17%); LCL injury, 4 (6%)	NR	Meniscal surgery: early ACLR, 69 (41%); delayed ACLR, 12 (40%) ACLR groups combined: meniscal excision, 21 (26%); meniscal repair, 45 (56%); meniscal trephination/rasping, 15 (18%)
Keays^30^ (2023)	ACLR: meniscal injury, 34 (61%) Rehabilitation: meniscal injury, 26 (58%)	NR	Meniscectomy: 34 (61%)
Kessler^31^ (2008)	NR	NR	NR
Kvist^33^ (2020)	ACL repair: meniscal injury, 34 (53%); cartilage injury, 4 (6%); MCL injury, 26 (41%) Delayed repair ≤2 y: meniscal injury, 16 (59%); MCL injury, 11 (41%) Delayed repair >2 y: meniscal injury, 5 (56%); MCL injury, 1 (11%) Rehabilitation: meniscal injury, 37 (70%); cartilage injury, 5 (9%); MCL injury, 21 (40%)	NR	Meniscal injuries: ACL repair, 21 (33%); delayed repair ≤2 y, 10 (37%); delayed repair >2 y, 3 (30%) MCL injuries: ACL repair, 17 (27%); delayed repair ≤2 y, 7 (26%); delayed repair >2 y, 1 (11%)
Lin^37^ (2017)	ACLR: meniscal injury, 665 (48.4%) Rehabilitation: meniscal injury, 835 (11.3%)	NR	ACLR: meniscal repair, 479 (34.9%) Rehabilitation: meniscal repair, 259 (3.5%)
Lu^38^ (2025)	ACLR: medial meniscal injury, 270 (27.7%); lateral meniscal injury, 227 (23.3%); medial + lateral meniscal injury, 157 (16.1%); PCL injury, 11 (1.1%); MCL injury, 161 (16.5%); LCL injury, 36 (3.7%); PLC injury, 27 (2.8%); patellar instability, 17 (1.7%) Rehabilitation: medial meniscal injury, 70 (31.8%); lateral meniscal injury, 29 (13.2%); medial + lateral meniscal injury, 28 (12.7%); PCL injury, 4 (1.8%); MCL injury, 28 (12.7%); LCL injury, 11 (5.0%); PLC injury, 3 (1.4%); patellar instability, 2 (0.9%)	NR	NR
Meuffels^40^ (2009)	NR	NR	Meniscectomy: ACLR, 17 (68%); rehabilitation, 20 (80%)
Meunier^41^ (2007)	ACL repair: meniscal injury, 22 (50%); MCL injury, 27 (61%) Rehabilitation: meniscal injury, 32 (57%); MCL injury, 25 (45%)	NR	Meniscectomy: ACL repair, 16 (36%); rehabilitation, 18 (32%) MCL repair: ACL repair, 18 (41%); rehabilitation, 14 (25%)
Mihelic^42^ (2011)	ACLR: medial meniscal injury, 10 (28%); medial + lateral meniscal injury, 13 (36%) Rehabilitation: medial meniscal injury, 6 (33%); medial + lateral meniscal injury, 5 (28%)	NR	ACLR: medial meniscectomy, 10 (28%); medial + lateral meniscectomy, 13 (36%) Rehabilitation: medial meniscectomy, 6 (33%); medial + lateral meniscectomy, 5 (28%)
Streich^62^ (2011)	NR	ACLR: preoperative IKDC grade A, 37 (92.5%); preoperative IKDC grade B, 3 (7.5%) Rehabilitation: preoperative IKDC grade A, 38 (95%); preoperative IKDC grade B, 2 (5%)	Partial meniscectomy: ACLR, 9 (23%); rehabilitation, 10 (25%)
Tsoukas^64^ (2016)	NR	NR	NR
van Yperen^66^ (2018)	ACLR: meniscal injury, 6 (24%)	NR	Meniscectomy: 6 (24%)
Wellsandt^69^ (2018)	ACLR: meniscal injury, 35 (42.1%); bone bruise, 59 (71.1%)	NR	NR
van Meer^65^ (2016)	NR	NR	Partial meniscectomy: 20 (21.5%)

a ACL, anterior cruciate ligament; ACLR, anterior cruciate ligament reconstruction; IKDC, International Knee Documentation Committee; LCL, lateral collateral ligament; MCL, medial collateral ligament; NR, not reported; OA, osteoarthritis; PCL, posterior cruciate ligament; PLC, posterolateral corner.
